# Dietary insulin index and load and cardiometabolic risk factors among people with obesity: a cross-sectional study

**DOI:** 10.1186/s12902-023-01377-4

**Published:** 2023-05-24

**Authors:** Mahdi Vajdi, Abnoos Mokhtari Ardekani, Zeinab Nikniaz, Babak Hosseini, Mahdieh Abbasalizad Farhangi

**Affiliations:** 1 Department of Community Nutrition, Student Research Committee, School of Nutrition and Food Sciences, Isfahan University of Medical Sciences, Isfahan, Iran; 2 Endocrinology and Metabolism Research Center, Institute of Basic and Clinical Physiology Science, & Physiology Research Center, Kerman University of Medical Sciences, Kerman, Iran; 3 Liver and Gastrointestinal Diseases Research Center, Tabriz University of Medical Sciences, Tabriz, Iran; 4 Department of Surgery, School of Medicine, Laparoscopy Research Center, Shiraz University of Medical Sciences, Shiraz, Iran; 5 Tabriz Health Services Management Research Center, Tabriz University of Medical Sciences, Attar Neyshabouri, Daneshgah Blv, Tabriz, Iran

**Keywords:** Metabolic syndrome, Obesity, Dietary insulin index, Dietary insulin load

## Abstract

**Background:**

The hypothesis of the effect of the insulinogenic effects of diet on the development of cardiometabolic disorders has been suggested, but limited data are available for adults with obesity. This study aimed to determine the association of dietary insulin index (DII) and dietary insulin load (DIL) with cardiometabolic risk factors among Iranian adults with obesity.

**Methods:**

The study was conducted with a total of 347 adults aged 20–50 years in Tabriz, Iran. Usual dietary intake was assessed through a validated 147-item food frequency questionnaire (FFQ). DIL was computed using published food insulin index (FII) data. DII was calculated by dividing DIL by the total energy intake of each participant. Multinational logistic regression analysis was performed to evaluate the association between DII and DIL and cardiometabolic risk factors.

**Results:**

Mean age of participants was 40.78 ± 9.23 y, and mean body mass index (BMI) was 32.62 ± 4.80 kg/m2. Mean of DII and DIL was 73.15 ± 37.60 and 196,242 ± 100,181. Participants with higher DII had higher BMI, weight, waist circumference (WC), and blood concentrations of triglyceride (TG) and Homeostasis model assessment insulin resistance index (HOMA-IR) (*P* < 0.05). After taking potential confounders into account, DIL was positively associated with MetS (OR: 2.58; 95% CI: 1.03–6.46), and high blood pressure (OR: 1.61; 95% CI: 1.13–6.56). Moreover, after adjustment for potential confounders, moderate DII was associated with increased odds of MetS (OR: 1.54, 95% CI: 1.36–4.21), high TG (OR, 1.25; 95% CI, 1.17–5.02), and high blood pressure (OR: 1.88; 95% CI: 1.06–7.86).

**Conclusion:**

This population-based study revealed that adults with higher DII and DIL associated with cardiometabolic risk factors and consequently, replacement of high with low DII and DIL may have reduce the risk of cardiometabolic disorders. Further studies with longitudinal design are required to confirm these findings.

## Introduction

Cardiovascular diseases (CVDs) are the leading cause of mortality worldwide and its treatment remains very challenging [1]. As highlighted in the literature, several factors such as obesity, hyperglycemia, insulin resistance, dyslipidemia, and hypertension play important roles in the origin of CVDs [2, 3]. While some risk factors of CVDs such as genetic, gender, and age are uncontrollable [4–6], there are behavioral risk factors such as low physical activity, smoking, obesity, and poor dietary habits, which account for more than 70% of the risk of CVDs [7–10]. Evidence is emerging that a healthy dietary pattern characterized by foods lower in refined sugars and starches and higher in dietary fiber, mainly soluble fiber, may be related to better health status, including better glucose control and lower CVD risk factors such as total cholesterol (TC), low-density lipoprotein cholesterol (LDL-C) and triglyceride (TG) [11, 12]. Given the role of diet, in CVDs, finding a dietary factor that might be involved in this condition is of great interest.

Nowadays it is well established that dietary carbohydrate is the main factor that impacts postprandial glucose levels and therefore plays an essential role in postprandial insulin secretion [13]. Several studies have revealed that high-carbohydrate diets, which cause high levels of blood insulin and glucose, directly related to insulin resistance, body fat stores, and unfavorable lipid profiles [14–19]. These diets have been shown to raise fasting TG levels, mainly by increasing hepatic synthesis of very low-density lipoprotein, and reduce high-density lipoprotein cholesterol (HDL-C) levels [20]. The insulinogenic effect of diet is usually estimated by the glycemic load (GL) and glycemic index (GI) [21]. The GI is a quantitative assessment of a food’s carbohydrate content according to their ability to raise postprandial blood glucose [21]. The consumption of high GI diets leads to postprandial hyperinsulinemia, which has been related to diabetes, obesity and other CVDs risk factors, such as increased TC, and TGs and reduced HDL-C levels [18, 22–24]. However, it should also be noted that dietary GI and GL does not reflect the effect of other dietary factors such as dietary protein and fats. Nevertheless, these factors produced a significant insulin response [25, 26].

Holt et al. [27] compared postprandial insulin responses of different foods and introduced insulin indices such as insulin load (DIL) and dietary insulin index (DII) for each food according to its insulinemic influence in relation to white bread as reference. While the GI provides valuable data about the glycemic potential of foods, DII determine the insulinogenic properties of several foods to measure the postprandial insulin response induced by fat, carbohydrate, and protein consumption, is more appropriate index compared with the GI for examining relations with several chronic diseases [27–31]. A cross-sectional study that investigated 203 overweight/obese adolescents has documented that higher DII and DIL were related to higher risk of metabolically unhealthy obese [32]. Moreover, a study among Iranian adults indicated that DII and DIL had no association with the risk of metabolic syndrome (MetS) [33]. Investigating the relationship of DII with chronic diseases is mainly related to obesity since stimulating more insulin release results in increased body fat mass by decreasing fat oxidation and elevating carbohydrate oxidation. Despite the relationship of DIL and DII with numerous chronic diseases reported in previous studies [27–29, 34], as far as we know, no study has investigated the association between DIL, DII, and cardiometabolic risk factors among adults with obesity. So, the aim of the current study was to assess the relationship between DII, DIL and cardiometabolic risk factors in a sample of adults with obesity.

## Materials and methods

### Study population

The current research is a cross-sectional study performed in 347 healthy adults (145 females and 202 males) aged 18–50 years, in Tabriz and Tehran cities of Iran. The sampling was performed using convenience method through announcements. In our study population, subjects with a BMI ≥ 30 kg/ m^2^ without prior history of drug or alcohol abuse, any presence of inflammations and infections, cardiovascular or renal disorders, hypertension, thyroid diseases, and diabetes mellitus were invited to participate in the study and were interviewed by a trained dietitian. Individuals of age < 18 years old or a history of weight change (> 5 kg) in the last 6 months, lactation and pregnancy were excluded from this study. To compute the sample size, the association between dietary quality indices and obesity was considered as a key dependent variable. The sample size calculation, using G-power software was based on the correlation coefficient (r) of 0.25, α = 0.05, and power 80%, the minimum sample size was 290 and considering 15% drop-out, the final sample size was 347 adults [35]. The study was received the approval of the ethics committee (Registration: IR.TBZMED.REC.1402.011) of Tabriz University of Medical Sciences. All participants signed the informed consent form.

### Demographic, anthropometric and biochemical assessment

General information about demographic characteristics (e.g., educational level, gender, history of smoking, marital status, and age) were obtained by using questionnaires by a trained dietitian using face-to-face interview. The IPAQ was used to measure the physical activity levels of the participants. All anthropometric measures were carried out on same day for each participant by an expert researcher. Body weight was measured using a Seca scale (Seca, Germany) with a sensitivity of 0.1 kg and height was measured to the nearest 0.1 cm, using a stadiometer. BMI was measured as weight divided by squared height (kg/m2). Waist circumference (WC) was measured in the standing position at midway level between lower rib margin and iliac crest using a constant tension tape to the nearest 0.1 cm. Waist Hip ratio was measured as WC divided by hip circumference. Fat mass (FM) and fat-free mass (FFM) were measured by BIA analyzer (Tanita, BC-418 MA, Tokyo, Japan). Systolic blood pressure (SBP) and diastolic blood pressure (DBP) were measured twice after the participants had been at rest for at least 15 min in the seated position by using a standardized mercury sphygmomanometer, and the average of the two measurements was used in all analyses. After 10–12 h of fasting, venous blood samples were taken into tubes between 7:00 am and 9:00 am. The samples were then separated by centrifugation at 4600 rpm for 15 min at 4 °C, and were frozen at -80 °C, in a freezer until analysis. HDL-C, TG, and TC serum levels were assessed by enzymatic method (Pars Azemoon Co., Tehran, Iran). Furthermore, LDL-C calculated using the Friedewald’s equation [36]. Serum levels of insulin were assayed using commercially available enzyme-linked immunosorbent assay kits (Bioassay Technology Laboratory, Shanghai City, China), in accordance with manufacturer’s instructions. Homeostatic model assessment for insulin resistance (HOMA-IR) and quantitative insulin sensitivity check index (QUICKI) were calculated using the formula.

### Dietary assessment

The validated semi-quantitative food frequency questionnaire (FFQ) of 147 food items was applied to collect data on dietary intake [37]. An expert nutritionist asked the study subjects to select the amount and frequency of the consumption of each food during the former year on an annual, monthly, weekly, or daily basis. Then, portion sizes of foods were also converted to gram using household measures. Because the Iranian Food Composition Table (FCT) provides a few data to analyze foods for nutrients and energy, we used the US Department of Agriculture (USDA) FCT.

### Calculation of DII and DIL

The food insulin index; for each food item was obtained from published studies developed by Holt et al. [27], Bell et al. [38], and Bao et al. [39]. The food insulin index is an algorithm for ranking foods and estimates the incremental insulin area under the curve (AUC) over two hours in response to the intake of a 1000-kJ portion of the test food divided by the AUC after intake of a 1000-kJ portion of the reference food. To calculate DIL, we first estimated the insulin load of each food using the following equation: Insulin load of a given food = insulin index of that food × energy content of that food (KJ/d). By summing up the insulin load of each food item, DIL was measured for each subject. Then, we computed the DII for each subject by dividing DIL by total energy consumption.

### Statistical analyses

Statistical analysis was performed using SPSS version 21.0 (Armonk, NY, IBM Corp) and *p*-values < 0.05) were used to show statistical significance. DIL and DII were adjusted for energy with the use of the residual method, before categorizing into quartiles. All subjects were categorized according to quartiles of DII and DIL scores. The first quartile of DII and DIL was taken as the reference category. Qualitative and quantitative variables were described as percentages and Mean ± SD, respectively. The X^2^ test was used to evaluate the distribution of categorical variables across quartiles of DII and DIL and One-way analysis of variance (ANOVA) was applied to compare the continuous variables across quartiles of DII and DIL. The multinomial logistic regression was applied to determine the association between quartiles of DII, DIL and cardiometabolic risk factors and also sex, age, education, physical activity level, marital status, BMI, and energy consumption as covariates.

## Results

### Participant characteristics

The mean ± SD age of the subjects (145 females and 202 males) was 40.78 ± 9.23 years and BMI was 32.62 ± 4.80 kg/m2. Mean of DII and DIL was 73.15 ± 37.60 and 196,242 ± 100,181. Baseline characteristics of the individuals across quartiles of DII and DIL are accessible in Table 1. Although most baseline variables were similar in the quartiles of DII and DIL, participants in the last quartile of DIL compared with participants in the first quartile were older and had higher intake of fat, carbohydrate, and protein (*P* < 0.05). Participants in the top quartile of DII compared with participants in the bottom quartile had higher BMI, weight, and higher intake of fat, carbohydrate, and protein (*P* < 0.05). As shown in Table 2, we did not observe any statistically significant difference in biochemical parameters across quartiles of DIL (*P* > 0.05). However, those in the top quartile of DII had higher WC, DBP and HOMA-IR concentration when compared with those in the bottom quartile (*P* < 0.05). Also, participants in the second quartile of DII had higher blood TG concentration when compared with those in the first quartile (*P* < 0.05).

**Table 1:** Baseline characteristics of study population across quartiles of energy-adjusted DIL and DII (*n* = 347)

	Quartiles of DIL					Quartiles of DII				
Variables	**Q1 (** ***N*** ** = 86)**	**Q2 (** ***N*** ** = 87)**	**Q3 (** ***N*** ** = 87)**	**Q4 (** ***N*** ** = 87)**	***P*-value**	**Q1 (** ***N*** ** = 86)**	**Q2 (** ***N*** ** = 87)**	**Q3 (** ***N*** ** = 87)**	**Q4 (** *N* ** = 87)**	***P*-value**
***Q*** **ranges**	< 154 8236	154 8236 − 187 882	187 882 − 232 719	> 232 719		< 47.88	47.88–69.56	69.56–96.11	> 96.11	
Age (years)	39.45 ± 9.68	40.69 ± 9.08	40.02 ± 8.36	43.03 ± 9.05	**0.05**	40.50 ± 9.14	39.02 ± 9.41	42.29 ± 9.35	41.31 ± 8.86	0.11
Gender (male )	54 (48%)	48(55.2%)	52(59.8%)	48(55.2%)	0.48	56(65.1%)	48(55.2%)	49(56.3%)	49(56.3%)	0.51
BMI(kg/m^2^)	33.11 ± 5.19	33.22 ± 5.40	32.53 ± 4.12	31.63 ± 4.30	0.11	31.82 ± 4.21	31.69 ± 4.66	33.33 ± 5.17	33.65 ± 4.89	**0.01**
Weight (kg)	93.63 ± 16.01	92.44 ± 15.54	92.41 ± 10.42	89.98 ± 15.09	0.40	89.51 ± 13.20	90.28 ± 14.21	93.83 ± 15.29	94.85 ± 14.50	**0.03**
FM (kg)	33.92 ± 10.63	34.25 ± 9.30	32.96 ± 7.52	34.11 ± 8.50	0.90	33.68 ± 7.19	34.00 ± 10.06	33.36 ± 9.32	34.01 ± 9.58	0.98
FFM (kg)	61.85 ± 13.17	61.50 ± 12.65	62.82 ± 11.40	63.37 ± 12.16	0.89	61.95 ± 12.52	61.78 ± 12.75	61.89 ± 11.46	63.33 ± 12.64	0.19
**Physical activity (%)**					0.49					0.81
Low	40(46.4%)	43(49.1%)	43(48.9%)	41(46.9%)		38(44%)	47(54.2%)	41(47.2%)	38(44.2%)	
Medium	27(32.1%)	28(32.1%)	15(17%)	24(28.15)		27(32%)	21(23.7%)	19(22.2%)	28(32.6)	
High	19(21.4%)	16(18.9%)	29(34%)	22(25%)		21(24%)	19(22%)	26.62(30.6%)	21(23.3%)	
**Marital status (%)**										
Married	68(79.1%)	75(85.1%)	78(89.7%)	76(87.4%)	0.67	66(76.7%)	76(87.4%)	76(87.4%)	78(89.7%)	0.31
**Education (%)**					0.84					0.06
Illiterate	1(1.8%)	1(1.9%)	0(0)	0(0)		7(8%)	0(0)	0(0)	0(0)	
≤ High school/diploma 25(28.5%)		20(22.7%)	17(19.2%)	14(16.2%)		19(22%)	28(32.2%)	35(40.3%)	18(21%)	
≥ College degree 60(69.7%)		66(75.5%)	70(80.9%)	73(83.9%)		60(70%)	59(67.8%)	52(59.7%)	69(79%)	
Carbohydrate (g)	468.43 ± 160.14	405.70 ± 164.94	432.80 ± 138.66	506.43 ± 199.98	**0.01**	337.63 ± 136.63	463.24 ± 113.53	404.07 ± 105.89	620.27 ± 180.96	**0.01**
Fat (g)	91.16 ± 38.15	93.68 ± 41.68	93.62 ± 32.90	125.92 ± 64.04	**0.01**	67.91 ± 29.86	104.69 ± 31.60	84.27 ± 23.97	150.01 ± 54.61	**0.01**
Protein (g)	100.81 ± 32.01	91.41 ± 35.48	100.11 ± 30.82	107.15 ± 47.90	**0.05**	77.01 ± 29.13	103.45 ± 25.55	90.74 ± 24.56	130.72 ± 45.04	**0.01**

*FM* Fat mass, *FFM* fat free mass, *BMI* body mass index

*P*-value obtained using one-way ANOVA for continuous variables and Chi-square test for categorical variables. Categorical and continuous variables data are presented as number (percent) and mean (SD). Bold values indicate *P* < 0.05 as the level of significance

**Table 2:** Anthropometric and biochemical measures across quartiles of energy-adjusted DIL and DII (*n* = 347)

	Quartiles of DIL					Quartiles of DII				
Variables	**Q1 (** ***N*** ** = 86)**	**Q2 (** ***N*** ** = 87)**	**Q3 (** ***N*** ** = 87)**	**Q4 (** ***N*** ** = 87)**	***P*** **-value**	**Q1 (** ***N*** ** = 86)**	**Q2 (** ***N*** ** = 87)**	**Q3 (** ***N*** ** = 87)**	**Q4 (** ***N*** ** = 87)**	***P*-value**
***Q *ranges**	< 154 8236	154 8236 − 187 882	187 882 − 232 719	> 232 719		< 47.88	47.88–69.56	69.56–96.11	> 96.11	
MetS (%)	37(43%)	26(29.9%)	36(41.9%)	42(48.3%)	0.08	35(40.7%)	26(29.9%)	42(48.3%)	38(44.2%)	0.08
WC (cm)	105.24 ± 9.00	106.35 ± 7.57	108.25 ± 10.99	107.30 ± 10.45	0.19	105.25 ± 8.04	105.47 ± 9.21	107.70 ± 10.30	108.72 ± 10.45	**0.04**
WHR	0.93 ± 0.06	0.94 ± 0.08	0.93 ± 0.07	0.92 ± 0.07	0.25	0.92 ± 0.08	0.93 ± 0.08	0.93 ± 0.07	0.94 ± 0.06	0.55
SBP (mmHg)	122.08 ± 15.40	124.72 ± 19.10	121.21 ± 13.21	123.95 ± 17.16	0.46	124.26 ± 16.96	119.41 ± 14.37	124.64 ± 18.68	123.67 ± 14.79	0.12
DBP (mmHg)	80.62 ± 11.02	83.51 ± 12.81	80.81 ± 11.29	82.14 ± 11.51	0.33	81.89 ± 11.83	78.82 ± 10.53	83.04 ± 13.33	83.35 ± 10.47	**0.04**
FBS (mg/dl)	92.52 ± 18.84	90.42 ± 12.11	91.91 ± 11.55	95.79 ± 28.83	0.30	92.08 ± 19.04	91.63 ± 12.50	95.20 ± 28.62	91.72 ± 11.65	0.56
TC (mg/dl)	187.47 ± 28.98	192.58 ± 39.29	192.26 ± 35.98	193.44 ± 41.36	0.70	186.51 ± 30.13	187.60 ± 36.65	199.02 ± 35.82	192.61 ± 42.22	0.09
TG (mg/dl)	151.18 ± 95.61	133.28 ± 62.06	158.84 ± 105.02	166.95 ± 105.48	0.10	134.55 ± 92.86	145.54 ± 71.22	152.51 ± 86.02	177.52 ± 116.52	**0.02**
LDL-C (mg/dl)	120.05 ± 27.33	125.99 ± 31.71	121.91 ± 30.72	126.71 ± 36.82	0.45	120.06 ± 26.59	120.86 ± 32.25	126.84 ± 31.81	126.97 ± 35.86	0.31
HDL-C (mg/dl)	42.29 ± 9.44	45.09 ± 8.56	43.06 ± 8.83	42.85 ± 10.97	0.23	42.63 ± 9.26	43.96 ± 8.71	42.21 ± 9.81	44.50 ± 10.21	0.34
Insulin (U/mL)	14.56 ± 9.17	15.65 ± 10.75	17.28 ± 12.28	17.99 ± 21.93	0.49	15.27 ± 12.75	15.95 ± 7.64	19.73 ± 21.41	14.17 ± 10.25	0.15
HOMA-IR	3.39 ± 2.27	3.55 ± 2.67	3.94 ± 2.61	4.41 ± 5.29	0.33	3.48 ± 2.80	3.66 ± 1.87	3.28 ± 2.72	4.79 ± 5.09	**0.05**
QUICKI	0.33 ± 0.03	0.33 ± 0.03	0.32 ± 0.03	0.32 ± 0.04	0.78	0.33 ± 0.03	0.32 ± 0.02	0.32 ± 0.04	0.33 ± 0.03	0.23

*Abbreviations:* *WC* Waist circumference, *WHR* Waist hip ratio, *TG* Triglyceride, *TC* Total cholesterol, *HDL-C* High-density lipoprotein cholesterol, *FBS* Fasting blood sugar, *SBP* Systolic blood pressure, *DBP* Diastolic blood pressure

*p*-value obtained using one-way ANOVA for continuous variables, and Chi-square test for categorical variables. Continuous variables are presented as the means ± standard deviations. Bold values indicate *P* < 0.05 as the level of significance

### Association between DII and DIL and cardiometabolic risk factors

Multivariable-adjusted odds ratios (ORs) for cardiometabolic risk factors across quartiles of DII and DIL have been indicated in Table 3. In the adjusted model 1, participants in the top quartile of the DIL tended to have 2.58-fold higher odds for MetS compared with participants in the lowest quartile (OR: 2.58; 95% CI: 1.03–6.46). Moreover, in adjusted model 1, we found that those in the top quartile of the DIL had the higher odds of high blood pressure compared with participants in the bottom quartile (OR: 1.61; 95% CI: 1.13–6.56). However, no significant association was observed between other cardiometabolic risk factors and DIL. No significant relationship was observed between DII and risk of MetS. Nevertheless, after adjusted potential confounders, participants in the top quartile of DII had greater risks for having MetS compared with those in the first quartile (OR: 1.54, 95% CI: 1.36–4.21). In the case of blood TG, in the unadjusted model (OR, 1.21; 95% CI, 1.15–5.78) and after adjustment for potential confounders (OR, 1.25; 95% CI, 1.17–5.02), those in the highest quartile of DII had significantly higher risks for higher TG levels compared with participants in the lowest quartile. Moreover, after adjustment for potential confounders such as age, gender, education, occupation, marital status, and physical activity, participants in the last quartile of DII had higher risks of elevated blood pressure compared with those in first category (OR: 1.88; 95% CI: 1.06–7.86), although this relationship did not remain after the full multivariate adjustment (OR: 1.52; 95% CI: 0.91–5.53).

**Table 3:** Odd ratio and 95% confidence interval for having different cardiometabolic risk factors according to quartiles of energy-adjusted DIL and DII (*n* = 347)

	Quartiles of DIL					Quartiles of DII				
Variables	**Q1 (*N* = 86)**	**Q2 (*N* = 87)**	**Q3 (*N* = 87)**	**Q4 (*N* = 87)**	***P*-value**	**Q1 (*N* = 86)**	**Q2 (*N* = 87)**	**Q3 (*N* = 87)**	**Q4 (*N* = 87)**	***P*-value**
***Q *ranges**	< 154 8236	154 8236-187 882	187 882-232 719	> 232 719		< 47.88	47.88–69.56	69.56–96.11	> 96.11	
**MetS**										
Crude	1 (Ref.)	1.77 (0.94–3.31)	1.04 (0.57–1.92)	1.80 (0.44–1.97)	0.08	1 (Ref.)	1.86 (0.47–3.58)	1.73 (0.40–2.34)	1.61 (0.85–3.02)	0.07
Model 1	1 (Ref.)	1.22 (0.44–3.53)	1.71 (0.66–4.41)	**2.58 (1.03–6.46)**	**0.04**	1 (Ref.)	1.36 (0.54–3.43)	1.99 (0.37–2.60)	**1.54 (1.36–4.21)**	**0.01**
Model 2	1 (Ref.)	2.59 (0.98–6.85)	2.66 (0.62–4.43)	2.14 (0.40–3.22)	0.06	1 (Ref.)	1.82 (0.22–3.04)	1.59 (0.16–2.12)	1.64 (0.90–4.72)	0.46
**High WC**										
Crude	1 (Ref.)	0.51 (0.08–2.90)	0.88 (0.18–4.17)	1.34 (0.28–6.42)	0.75	1 (Ref.)	1.13 (0.24–5.35)	0.92 (0.14–5.81)	1.17 (0.22–6.14)	0.99
Model 1	1 (Ref.)	0.57 (0.09–3.36)	1.04 (0.20–5.34)	1.46 (0.29–7.32)	0.79	1 (Ref.)	1.04 (0.20–5.28)	0.92 (0.14-6.00)	1.22 (0.22–6.62)	0.99
Model 2	1 (Ref.)	2.10 (0.91–4.85)	1.15 (0.48–2.75)	1.32 (0.52–3.36)	0.31	1 (Ref.)	1.07 (0.15–7.28)	1.27(0.12–13.58)	1.77 (0.17–18.21)	0.95
High Cholesterol										
Crude	1 (Ref.)	0.60 (0.28–1.35)	0.68 (0.29–1.60)	0.61 (0.24–1.54)	0.60	1 (Ref.)	1.27 (0.56–2.86)	0.81 (0.33–1.97)	0.92 (0.39–2.19)	0.76
Model 1	1 (Ref.)	0.67 (0.29–1.56)	0.68 (0.27–1.70)	0.63 (0.24–1.67)	0.74	1 (Ref.)	1.24 (0.53–2.91)	0.84 (0.33–2.12)	0.93 (0.38–2.29)	0.85
Model 2	1 (Ref.)	0.64 (0.26–1.56)	0.68 (0.26–1.74)	0.64 (0.24–1.72)	0.74	1 (Ref.)	1.30 (0.49–3.41)	0.74 (0.23–2.40)	0.77 (0.22–2.60)	0.56
High TG										
Crude	1 (Ref.)	0.96 (0.32–2.87)	1.13 (0.41–3.13)	2.19 (0.96–8.15)	0.16	1 (Ref.)	2.00 (0.75–5.29)	1.41 (0.54–3.68)	**1.21 (1.15–5.78)**	**0.02**
Model 1	1 (Ref.)	0.85 (0.32–2.26)	1.06 (0.42–2.63)	2.80 (0.28–5.94)	0.29	1 (Ref.)	1.90 (0.67–5.39)	1.33 (0.46–3.87)	**1.25 (1.17–5.02)**	**0.02**
Model 2	1 (Ref.)	0.93 (0.30–2.82)	1.02 (0.36–2.89)	2.47 (0.82–7.45)	0.90	1 (Ref.)	1.10 (0.26–4.72)	1.81 (0.20–3.28)	2.56 (0.84–5.83)	0.21
**Low HDL-C**										
Crude	1 (Ref.)	1.73 (0.81–3.70)	1.01 (0.45–2.23)	1.26 (0.52–3.03)	0.46	1 (Ref.)	1.23 (0.57–2.62)	0.81 (0.33–1.93)	1.27 (0.55–2.89)	0.72
Model 1	1 (Ref.)	1.80 (0.81–3.96)	1.01 (0.43–2.36)	1.28 (0.51–3.19)	0.43	1 (Ref.)	1.28 (0.58–2.80)	0.76 (0.30–1.88)	1.23 (0.52–2.88)	0.65
Model 2	1 (Ref.)	2.10 (0.91–4.85)	1.15 (0.48–2.75)	1.32 (0.52–3.36)	0.31	1 (Ref.)	1.76 (0.70–4.38)	1.16 (0.37–3.64)	2.08 (0.65–6.65)	0.41
Hyperglycemia										
Crude	1 (Ref.)	1.19 (0.45–3.16)	0.77 (0.30-2.00)	0.46 (0.17–1.24)	0.30	1 (Ref.)	2.34 (0.84–6.51)	2.63 (0.24–5.83)	1.89 (0.34–5.29)	0.07
Model 1	1 (Ref.)	1.49 (0.53–4.14)	0.79 (0.28–2.21)	0.49 (0.17–1.41)	0.23	1 (Ref.)	2.09 (0.72–6.07)	1.60 (0.22–5.64)	1.87 (0.32–5.34)	0.14
Model 2	1 (Ref.)	1.33 (0.46–3.88)	0.73 (0.25–2.10)	0.46 (0.15–1.33)	0.24	1 (Ref.)	2.20 (0.55–2.84)	1.33 (0.40–5.45)	1.27(0.36–5.15)	0.12
Elevated blood pressure										
Crude	1 (Ref.)	1.02 (0.39–2.68)	2.73 (0.97–7.69)	1.52 (0.63–3.68)	0.19	1 (Ref.)	1.18 (0.44–5.19)	1.13 (0.40–5.19)	1.47 (0.86–7.10)	0.33
Model 1	1 (Ref.)	1.31 (0.45–3.78)	2.13 (0.81–5.62)	**1.61 (1.13–6.56)**	**0.03**	1 (Ref.)	1.13 (0.45–5.84)	1.01 (0.38–5.62)	**1.88 (1.06–7.86)**	**0.03**
Model 2	1 (Ref.)	1.21 (0.40–3.65)	3.59 (0.10–5.21)	1.15 (0.75–6.19)	0.15	1 (Ref.)	1.53 (0.12–5.39)	1.56 (0.13–5.37)	1.52 (0.91–5.53)	0.34

Model 1: Adjusted for age, gender, education, occupation, marital status, and physical activity. Model 2: Model 1 + energy intake and BMI

*Abbreviations:* *DIL* Dietary insulin load, *DII* Dietary insulin index, *HDL-C* High-density lipoprotein-cholesterol, *WC* Waist circumference

*P*-values are reported based on the logistic regression test and are considered significant at ˂0.05. Bold Values indicate *P* < 0.05 as the level of significance

## Discussion

To our best of knowledge, the current cross-sectional study is the first try to focus on the relationship of DIL and DII assessed by FFQ and cardiometabolic risk factors among Iranian adults with obesity. Our results revealed that a higher DIL score might be positively related to greater risks of MetS, and higher blood pressure. Also, a higher DII score might be related to higher odds of MetS, higher TG concentrations, and higher blood pressure. Furthermore, there was no significant relationship between DII, DIL and higher WC, higher TC, and lower HDL-C either after or before taking potential covariates into account. Individuals with a higher DII score had higher BMI, weight, WC, blood concentrations of TG, and HOMA-IR.

**Fig. 1:**
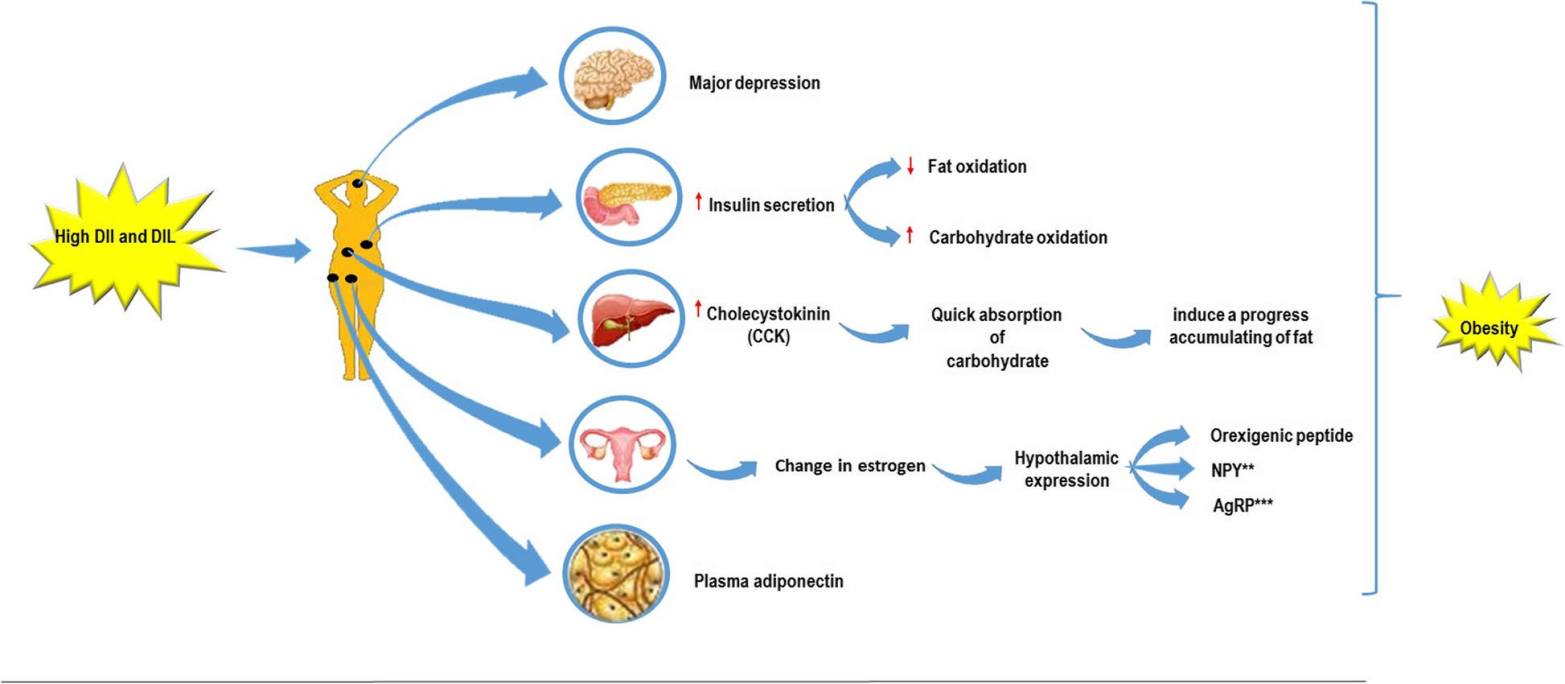
The mechanistic pathways of dietary DII and DIL in developing obesity and obesity related co-morbidities

Notwithstanding different approaches for assessing the insulinemic potential of foods, DII and DIL have attracted much international attention in recent decade [27, 39]; results from several studies have revealed that these indices are more truthful insulin response predictors to a mixed meal than other approaches [27, 40, 41]. Previous observational studies have examined the relationship between DIL and DII, cardiometabolic risk factors, type 2 diabetes mellitus, CVD, MetS, but the results of these studies has been controversial [42–44]. This may be due to that insulin resistance status and BMI seemed to modify the relationship between DIL, DII, and metabolic disorders [44, 45]. Nevertheless, no study has so far explored DII or DIL in relation to cardiometabolic risk factors as an outcome among Iranian adults with obesity. Insulin resistance and its associated abnormalities are important risk factor for development of complication in people with obesity so early detection and intervention are important, previous studies have reported that a diet that increased insulin level may increase metabolic risk factors [46, 47]. In the current study, significant positive relations were observed between DII and DIL and risk of MetS and higher blood pressure. This may be the result of oscillations of insulin secretion which is controlled by several factors including neuronal inputs, hormones, and nutrients [39, 48]. Several mechanisms have been proposed to explain the association of DII and DIL with risk of metabolic diseases. A diet with high DII and DIL may increase central obesity by reducing insulin sensitivity, which may, in turn, decrease lipolysis, resulting in augmented fat storage and therefore an increased risk of MetS [49]. Furthermore, as potentially high insulinemic foods have a high rate of absorption, digestion, and conversion to glucose, these foods would quickly increase the blood glucose and blood insulin and, therefore, decrease glucose excursion [50]. Anjom-Shoae et al. [44] showed no significant relationship between DII, DIL, and risk of obesity. Hyperinsulinemia has proposed increasing sympathetic nervous system activity, which increases heart rate, sodium retention, cardiac output, and consequently blood pressure [51]. Indeed, diets with high fructose are related to higher blood pressure and increases in sympathetic tone [52, 53], whereas increased glucose consumption is also related to increases in basal heart rate [54]. In addition, a meta-analysis by Evans et al. [55] showed that a reduction in GI was effective at reducing SBP and DBP. Some of the underlying mechanistic pathways are illustrated in Fig. 1.

We found that higher DII was meaningfully associated with higher risk of higher TG, even after adjustment for several covariates. Earlier studies have reported that GL or GI positively predict plasma TGs [56–58]. In line with our study, Nimptsch et al. [59] shown that DII was not related to glycated hemoglobin, and LDL-C, but a significant relationship was seen between DII with TG levels and HDL-C levels. In another study, by Joslowski et al. [60] a higher DIL and DII were related to higher body fat % in adulthood. Furthermore, two clinical trials reported that the diets with low GI had favorable effects on MetS components [61, 62]. It is proposed that a high DII and DIL during a long time period may increase insulin growth factor-1 and insulin secretion that may lead to body fat formation [60]. Low fat and high carbohydrate consumption have been related to higher TG and lower HDL-C levels [63, 64]. Another study reported that DII was related to enhance postprandial blood glucose without increased odds of hypoglycemia [65]. Nevertheless, a cross-sectional study found no relationship between the DII and glycemic control in adults [59]. The inconsistency might be attributed to different tools used for assessment of dietary intake, different confounders, and different study populations. Moreover, different food processing conditions in different cultures can be additional cause for the inconsistency. Intake of bread and rice that are insulinogenic foods is high in Iranian population and may another reason for this difference. Iranians receive approximately 62% of their energy intake from carbohydrates, which is significantly higher than other populations [66]. High insulinemic foods cause rapid rise and reduction in blood insulin and glucose [50]. This can decrease satiety and increased risk of obesity [67]. Mean DII and DIL scores in the present study were 73.15 and 196,242, respectively. DII scores in this study are close to other studies. For example, in the study by Nimptsch et al. [59] median DII was 42.8 in women and 41.7 in men. Nevertheless, DIL scores are much higher than other similar studies. This difference can be described by different approaches to calculate DII and DIL scores in different studies. For instance, similar studies used each serving of food while in this study; each gram of a given food was used in standard formula to calculate DII and DIL.

### Strengths and limitations

Several strengths of this study include an adjustment for several potential covariates in the analysis, being the first study among adults with obesity, and using a validated FFQ for constructing DII and DIL. However, several limitations should be noted. The present study was carried out in a cross-sectional design, which is prone to misclassification, selection bias, and response bias and would not allow us to infer causality. Although we used a validated FFQ, reporting of dietary consumption can still be subject to measurement error and recall bias. Therefore, the results of this study may not represent all adults with obesity. Although we have adjusted for several lifestyle factors, it is possible that mistakes in evaluating covariates may have led to residual confounding of the relations. Moreover, we did not observe a significant association between DII and DIL and odds of high WC, which may be due to the study only included obese individuals. Finally, in this study, LDL-C was calculated using Friedewald’s formula. This may subsequently leads to underestimation of calculated LDL-C.

## Conclusion

In conclusion, this population-based study revealed that adults with higher DII and DIL associated with cardiometabolic risk factors such as MetS, high TG, and hypertension and consequently, replacement of high with low DII and DIL may have reduce the risk of cardiometabolic disorders. Further studies with longitudinal design are required to confirm these findings.

## Data Availability

All of the data are available with reasonable request from the corresponding author.
